# Gender differences in reading and numeracy achievement across the school years

**DOI:** 10.1007/s13384-022-00583-8

**Published:** 2022-10-21

**Authors:** Damon P. Thomas, Belinda Hopwood, Vesife Hatisaru, David Hicks

**Affiliations:** 1 School of Education, University of Queensland, Brisbane, QLD 4072 Australia; 2 School of Education, University of Tasmania, Launceston, TAS 7248 Australia

**Keywords:** Assessment, Gender, Standardised testing, Reading, Numeracy

## Abstract

Developing students’ reading and numeracy skills remain key goals of contemporary schooling. In Australia, the National Assessment Program – Literacy and Numeracy (NAPLAN) tests have assessed these skills since 2008. Previous research found a significant gender gap in favour of females for the NAPLAN writing test, yet no study has examined whether gender gaps exist for reading and numeracy or their developmental pattern across the school years. Given the educational and public interest in NAPLAN and its considerable costs, it is important to understand what these tests reveal about student outcomes. The paper presents the first investigation of patterns of male and female student achievement on the NAPLAN reading and numeracy tests from 2008 to 2021. It applies the equivalent year level technique to explain the pedagogical significance of NAPLAN achievement and compares the findings with the writing gender gap to present a fuller picture of male and female achievement.

## Introduction

In Australia, the National Assessment Program – Literacy and Numeracy (NAPLAN) tests were introduced in 2008. These tests, which are completed by more than one million Australian students each year, seek to determine whether students are developing the reading, numeracy, writing, spelling, and grammar and punctuation skills that are deemed essential for a productive, successful adult life (Australian Curriculum, Assessment and Reporting Authority (ACARA), 2016d). Every year, the NAPLAN test is completed by all Australian students in Years 3, 5, 7 and 9, although the test was cancelled in 2020 due to COVID-19.

A range of unintended side effects of NAPLAN testing has attracted considerable negative attention to the assessment program. A key concern relates to the impact of NAPLAN testing on classroom practices. For example, research by Gannon (2019) and Ryan et al. (2021) and several others investigated the impacts of NAPLAN on the teaching and learning of literacy and numeracy, with many teachers across Australia devoting a disproportionate amount of class time each year to specific test preparation. NAPLAN testing has been associated with teaching experiences geared towards test taking, higher anxiety levels for teachers and students, and strong influences over policymaking across educational contexts (e.g. Hardy & Lewis, 2018; Lewis & Hardy, 2015). In fact, almost all research about NAPLAN testing has sought to shine a light on how the program has affected school processes in negative ways.

This situation is problematic for two reasons. First, curriculum and policy documents present clearly different perspectives. For example, according to the Melbourne Declaration on Educational Goals for Young Australians (2008) policy document, “schools need reliable, rich data on the performance of their students because they have the primary accountability for improving student outcomes” (p. 16), and the NAPLAN reports can provide schools with that data (e.g. Jackson, 2022). The more recent Alice Springs (Mparntwe) Education Declaration document published by the Education Council (2019) continues to emphasise value in using data to improve educational experiences and outcomes of students, such as evaluating the effectiveness of teaching practices and identifying students’ progress and growth. These perspectives have been often reiterated in ACARA’s communications. For example, a recent infographic (ACARA, 2022) outlined the utility of NAPLAN data for forward planning and tracking the progress and achievement of learners over the course of their educational experience (both individual and entire group levels). Secondly, Australian governments and school systems are investing millions of dollars and significant resources to undertake NAPLAN testing each year. While not discounting the problems associated with NAPLAN, there is need for more research exploring what testing at this immense scale can tell us about the literacy and numeracy achievement of different groups of learners.

### Drawing on NAPLAN test results to make informed decisions about teaching and learning

While it is often considered that the NAPLAN data “provide powerful diagnostic information which can be used to complement school assessment and to inform the planning of teaching and learning programs” (Victorian Curriculum & Assessment Authority, 2013, p. 2), to date, almost no research has drawn on the test results to make sense of practical issues concerning key areas of literacy and numeracy. A notable exception to this was the first longitudinal investigation into male and female achievement on the NAPLAN writing test across the tested year levels from 2011 to 2019 (Thomas, 2020). Thomas (2020) found a large gender gap favouring female students, stretching from just over eight months of equivalent learning in Year 3 to just over 24 months (2 years) in Year 9. In other words, the average Australian Year 9 male wrote texts of a similar quality to the average female in Year 7. This reflected similar findings from international writing assessments (Adams & Simmons, 2019; Reilly et al., 2019), yet Thomas’s research was the first to show the developmental trajectory of the writing gender gap, which increased across the tested year levels, but which increased most rapidly between Year 5 and Year 7 as students transitioned from primary to secondary school.

While students complete NAPLAN tests in reading, writing, spelling, grammar, and punctuation, and numeracy, to date, no longitudinal study has investigated male and female student achievement on any of these tests other than Thomas’s (2020) study on the writing test. Understanding patterns of student achievement is critical to targeting subsets of students who are in most need of additional support. Given the public interest in NAPLAN, the significant investment in funding and school resources each year, the troubling implications for teacher and student practices and wellbeing (Wyn et al., 2014), and misalignments between perspectives among teachers (e.g. Evans et al., 2021) and presented in curriculum and policy documents mentioned earlier, it is important to learn what we can from these tests to promote improved learning outcomes for all students. This paper addresses this gap by investigating patterns of male and female student achievement on the NAPLAN reading and numeracy tests from 2008 to 2021. It applies the Grattan Institute’s (Goss & Sonnemann, 2016) equivalent year levels technique to explain the pedagogical significance of NAPLAN achievement and compares the findings with the writing gender gap to present a more comprehensive picture of male and female literacy and numeracy achievement as measured by Australia’s only large-scale assessment.

The study will address the following research questions:

1. How have male and female students performed on the NAPLAN reading and mathematics tests between 2008 and 2021?
2. What is the pedagogical significance of these findings in terms of equivalent year levels?
3. How might these results be considered alongside the writing gender gap to offer a fuller account of male and female students’ literacy and numeracy achievement?

## Literature review

Reading and numeracy skills are amongst the most important to develop throughout an individual’s schooling. These skills are basic to educational, professional, and social success (Berman, 2009). Advances in artificial intelligence and digital and physical technologies have meant fewer unskilled jobs are available, making the development of reading and numeracy skills an important priority for every learner (Graham & Herbert, 2011). At school, students with strong reading and numeracy skills can understand different text forms easily and deeply, identify and recall the most relevant information, and make inferences between key points (Neufeld, 2006; Smith et al., 2021). More broadly, the health of a society and its economy rely on its citizens having sufficient literacy and numeracy skills to take advantage of individual and social opportunities (Caponera et al., 2016). Those who do not develop reading and numeracy skills at school are far more likely to have persistent issues in these areas as adults, and to be at risk of welfare dependence and physical and mental health problems (Partanen & Seigel, 2014). Those who do develop these skills, however, are more likely to pursue university studies and earn higher salaries, and these long-term impacts provide additional life, socioeconomic, and career benefits to female students in particular (Chetty et al., 2014). The importance of these skills is well accepted, which accounts for the considerable time dedicated to literacy and numeracy in essentially every school in the world.

This section discusses previous research that has investigated reading and numeracy outcomes of students. It presents a brief overview of the importance of reading and outlines gender differences shown by reading assessments nationally and internationally, before repeating this structure for numeracy. The section ends by mentioning potential factors and norms that have been suggested to influence student reading and numeracy achievement.

## Reading

### The importance of reading

Reading is arguably one of the most important and fundamental skills a child will learn in their first years of formal education. Reading plays a central role for learning in various domains, and for active participation in cultural and societal activities (Hochweber & Vieluf, 2018). Yet for some students, engaging in the process of reading can pose a number of challenges. While such challenges are experienced by both males and females, there is a wealth of research evidence to suggest that boys are more likely to experience challenges in reading than girls. This is evidenced in both the research literature (see Hek et al., 2019; Hochweber & Vieluf, 2018; Khorramdel et al., 2020; Logan & Johnston, 2010; Lynn & Mikk, 2009; Reilly et al., 2019), and results from national and international tests such as NAPLAN, the Program for International Student Assessment (PISA), the US National Assessment of Educational Progress (NAEP) and the Test of Reading Comprehension (TORCH). Such gender disparities in reading do not always exist of course, but when they are evident it is easy to summarise; males do better in numeracy and females do better in reading (Cobb-Clark & Moschion, 2017).

### Gender differences in reading assessments

Gender differences in reading tests are not a new revelation. From as early as 1910, studies have found that male students consistently score poorer on reading tests than female students (Pauley, 1951; Pickle, 1998) and show less interest and motivation to read (Logan & Johnston, 2010; McGeown et al., 2012). These test scores and attitudes towards reading become evident when students enter the primary years of schooling and continue into the secondary years of education (Mckenna et al., 2012).

Gender differences in reading performance on national and international large-scale assessments are regularly observed across countries. In PISA, which assesses the achievement of 15-year-old students, large gender gaps have appeared consistently (Khorramdel et al., 2020). In the 2018 PISA, for example, the gender gap for reading showed an effect size of 0.30 (Cohen’s *d*) (OECD, 2020), with females outperforming males. Similarly, in the 2016 Progress in International Reading Literacy Study (PIRLS) assessment, females showed a higher average achievement than males (Cohen’s *d* effect size 0.19). Similar patterns of overall difference between males and females in reading proficiency have been found in other large-scale assessments such as the NAEP and NAPLAN. While they only looked at performance on the Year 3 NAPLAN test, Cobb-Clark and Moschion’s research (2017) revealed that female students from low to middle socioeconomic status (SES) families had an advantage in reading over males, scoring higher on tests of reading comprehension.

Findings from national and international studies suggest a gender gap with reading will be apparent in a longitudinal exploration of NAPLAN results and, as with the writing gender gap (Thomas, 2020), the gap with reading is likely to increase between Year 3 and Year 9. A study by Scheiber et al. (2015) in the United States found that the gender gap for reading was approximately half the size of the gap for writing, with both gaps being in favour of females. This study seeks to provide a detailed description of the extent and developmental trajectory of a reading gender gap, to compare this with the writing gender gap (Thomas, 2020), and to discuss the pedagogical significance of this gap in terms of equivalent year levels.

## Numeracy

### The importance of numeracy

Numeracy can be described as a key competency in contemporary societies that is necessary for productive citizenship and employment. Many English-speaking countries, including Australia, consider the development of numeracy skills to be critically important, placing it at one of the highest policy levels (Goos et al., 2011). However, there is no widespread consensus over the definition of the construct *numeracy*. According to the ACARA (2017), numeracy involves “the knowledge, skills, behaviours and dispositions that students need to use mathematics in a wide range of situations,” and this requires students to “recognise and understand the role of mathematics in the world and have the dispositions and capacities to use mathematical knowledge and skills purposefully” (para. 3). The OECD (2012) defined numeracy as “the ability to access, use, interpret, and communicate mathematical information and ideas, in order to engage in and manage the mathematical demands of a range of situations in adult life” (p. 36). To that end, numeracy is about the use of mathematics in and on the world (Goos et al., 2011).

Students become numerate as they gain knowledge and skills to use mathematics confidently across subjects at school and outside the school (ACARA, 2017). Students who are numerate have mathematical knowledge, hold positive dispositions towards mathematics, use mathematical tools effectively, and use mathematical thinking in a range of context to analyse situations and draw conclusions (Goos et al., 2011). Despite its significance, there is evidence that many Australian 15-year-old students do not have adequate numeracy skills (e.g. Thomson et al., 2013) making them less prepared for tertiary studies, active citizenship, and employment.

### Gender differences in numeracy assessments

Comprehensive reviews of the research literature concerned with differences in the score achieved by male and female students on the NAPLAN test, and on international large-scale tests such as PISA and the Trends in International Mathematics and Science Study (TIMSS) are widely presented in previous research (e.g. Leder & Forgasz, 2018). In this section, we provide a brief overview of the relevant studies regarding gender differences in mathematics performance in NAPLAN and potential factors behind the existing gap. Overall, the findings highlight that gender gaps in mathematics achievement continue to be replicated—a larger percentage of males correctly answer the questions than females—and call for more recent studies.

Based on the results of the analyses of data from TIMSS in 2006, and PISA in 2006 and 2009, showing a considerable decline in females’ mathematics performance, Hill (2011) analysed the mathematics achievement of Grades 3, 5, 7, and 9 female students in NAPLAN data for the three years (2008 to 2010) to determine whether a similar decline was evident. Consistent with the results of analyses of the TIMSS and PISA data, the results of the analysis of NAPLAN data showed that females’ mathematics achievement in Australia is on a decline. Forgasz and Hill (2013) reported the NAPLAN data for 2008, 2009, and 2010 showing that for each year, in each state/territory, for students at Grades 3, 5, 7, and 9, on average, males outperformed females. Furthermore, gender inequalities observed in students’ test scores widen as students’ progress through their schooling (Hill, 2011), and are larger among high-performing students than low-performing students. Reviewing the NAPLAN National Report for 2016, Leder and Forgasz (2018) reported:

At the Year 3 level a slightly higher proportion of girls (96.0%) performed at or above the national minimum level compared with that of the boys (95.1%). Yet there was a higher proportion of boys (17.1%) than girls (12.7%) whose score placed them in the highest category available. Similarly, for students at the Year 9 level, a slightly higher proportion of girls (95.7%) than boys (94.7%) were deemed to have performed at or above the national minimum level. But at that year level too, a higher proportion of boys (9.7%) than girls (6.6%) recorded a score that placed them in the highest category. (p. 690)

The authors noted the persistent pattern of males outperforming females at each tested year level based on numeracy mean scores. This reflects persistent gender differences in mathematics performance that have been found in the broader literature beyond standardised assessment tests (e.g. Kane & Mertz, 2012). While Leder and Forgasz (2018) dedicated a section of their investigation into the validity of several standardised numeracy/mathematics tests to NAPLAN, they did not consider changes in the gender gap over time in detail. They included a figure showing male and female student achievement on the NAPLAN numeracy test between 2008 and 2016, which allows readers to easily compare student NAPLAN scores in Years 3, 5, 7, and 9. A comparison of NAPLAN scale scores only does not take into account the non-linear rate of student progress across the tested year levels. As discussed by Goss and Sonnemann (2018), students in the primary age groups typically make more progress between NAPLAN tests in terms of NAPLAN scale scores gains than those in the secondary age groups. This was a key reason for the authors to introduce the equivalent year levels approach as a more accurate way to interpret student NAPLAN achievement (Goss & Sonnemann, 2016). As outlined in the method section, the present study used the equivalent year level approach to show in more detail how the numeracy gender gap has changed across the tested year levels.

## Potential factors influencing gender differences in reading and numeracy achievement

While researchers have investigated some of the biological factors that enable male or female students to develop reading or numeracy skills (e.g. Berninger et al., 1996), this section focusses on some of the contextual factors (e.g. home, school, or broader environment) and affective variables (e.g. beliefs, views, emotions, attitudes) that directly or indirectly facilitate or inhibit student learning, and accordingly test performance. Such factors have been outlined in the research literature to explain persistent gender gaps in reading that favour female achievement and in numeracy that favour male achievement.

Gender stereotypes have been found to influence students’ perceived abilities and motivations for both reading (e.g. Khorramdel et al., 2020) and numeracy (e.g. Carmichael, 2014). Of concern is the view that gender stereotypes generally advantage males (Leder & Forgasz, 2018). Studies have shown that a considerable proportion of Australian adults believe that males are better at mathematics than females (Leder & Forgasz, 2011) and females are better at English than males (Leder et al., 2014). This is problematic since parental perceptions of children’s abilities and their expectations directly influence student reading and numeracy test outcomes (Carmichael, 2014). As an example, Carmichael (2014) found that Year 3 males whose parents expected them to pursue university studies performed better in numeracy than females whose parents held the same expectations. Hatisaru’s (2021) investigation into school students’ career interests across male and female students has revealed that, in contexts where parents’ academical expectations from daughters are higher, female students show clear interest in pursuing mathematical or related careers such as computer engineer, astronaut, and mechanical engineer. In a large-scale drawing-based study, Hatisaru (2020) found that both female and male primary school students predominantly depicted male mathematicians, and compared to males, female students were more likely to view mathematicians as male.

Students’ perceptions of mathematics and mathematicians develop throughout their years in school and are impacted by school-related factors, along with other factors such as family- or society-related factors. Student stereotypical perceptions of mathematics begin with exposure to different cultural and societal stereotypes via television, cartoons, books, and other media, and also via peers and adults through the repetition of negative phrases. In schools, students often experience direct teaching methods and do not see many applications of mathematics, which contributes to student perceptions of mathematics and mathematicians (Picker & Berry, 2000).

Teacher perceptions of male and female student abilities are another important consideration. A study by Leder et al. (2014) found that teacher perceptions were a key factor influencing student achievement in both reading and numeracy learning outcomes, including achievement, participation, and attitudes. As might be expected, students with poor attitudes towards reading or numeracy show less motivation for these areas, which directly impacts their participation and overall achievement (Khorramdel et al., 2020). In spite of not the focus of this study, strategies for addressing teacher perceptions and practices that can cause gender inequalities have been suggested by Pinkett and Roberts (2019).

An additional factor that can influence student reading and numeracy achievement is the style of test questions. Focussing on large-scale mathematics tests including PISA, TIMSS, and NAPLAN, Leder and Forgasz (2018) questioned whether these tests are gender neutral. The authors highlighted examples in their research, revealing that these tests’ content domains (e.g. number, geometry, probability) and response formats (e.g. free response, multiple choice, type of technology used) can impact student results. As explored by Oam (2015), tests that include multiple-choice style items might advantage males over females, since females generally take less risks than males. However, this suggestion does not explain why males have typically performed higher than females with numeracy, but lower with reading. As suggested by Cobb-Clark and Moschion (2017), “despite the multitude of explanations put forward for the gender gap in educational achievement it is fair to say that the literature has been better at documenting its existence than explaining its source” (p. 5). Clearly, further research is needed to unpack why the gender gap exists.

## The present study

As a precursor to more explanatory research, descriptive research is needed that clearly details patterns in student reading and numeracy achievement across the school years (Lee & Al Otaiba, 2015). Since test scores are often represented by seemingly arbitrary numbers, such research needs to be able to make these scores relevant pedagogically to help show the extent of any gender gaps over time. This study was designed to provide such a detailed account of male and female students’ reading and numeracy achievement between 2008 and 2021.

## Methodology

### The NAPLAN reading test

The NAPLAN reading test has concentrated on reading written English since it was introduced in 2008. This is despite calls from Unsworth et al. (2019) and others for the test to broaden its scope to reading multimodal text forms. As explained by ACARA (2016a), students completing the traditional, paper-based reading test are given a magazine with a variety of texts that demonstrate various written genres and are required to read the materials and fill out a separate booklet with associated questions. The test begins with basic, short texts and progresses to longer and more difficult texts to cater for different reading skills within each year level. The online reading test involves a variety of multiple-choice, short answer, and technology-enhanced questions, such as drag and drop. The online *tailored* test adapts to student reading skills, providing more or less difficult questions depending on previous answers. ACARA (2016b) argued that this adapting test “results in better assessment and more precise results” (para. 3). All Australian schools are expected to complete the online version of the reading test by 2022.

### The NAPLAN numeracy test

**Fig. 1:**
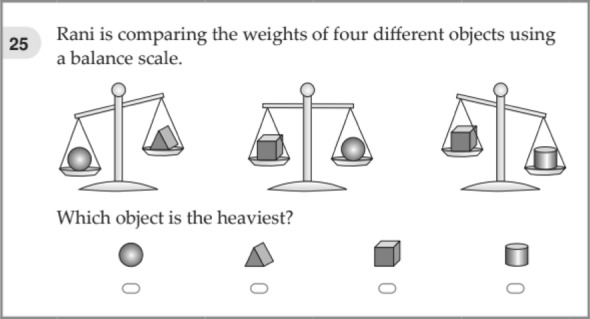
Example NAPLAN Year 3 numeracy test question

The NAPLAN numeracy test assesses the four proficiency strands (understanding, fluency, problem solving, and reasoning) across the three content domains of mathematics (number and algebra; measurement and geometry; and statistics and probability). It is closely aligned with the Australian Curriculum: Mathematics (AC:M) and has been described as a mathematics achievement test measuring AC:M learning areas (Leder & Forgasz, 2018). The paper-based and online numeracy tests involve multiple-choice and constructed response questions, while the online test also includes technology-enhanced questions. The Year 7 and Year 9 numeracy test includes a short non-calculator section with eight questions. The rest of the test can be completed with calculators. Test questions are often communicated through written words and images, as in the example from the Year 3 test in 2016 (ACARA, 2016c) (see Fig. 1).

#### Scoring NAPLAN

NAPLAN scale scores are reported for the five key domains: reading, writing, numeracy, spelling, and grammar and punctuation. Each domain is scored on a scale which ranges from 0 to 1000 and covers all tested year levels. While these scores are designed to have the same meaning over time (i.e. scoring 550 on the reading test in Year 3 means the same outcome in 2008 and 2018), interpretation of these scores across year levels is difficult, beyond determining whether or not a student is at, above, or below the average (or minimum standard) for a given year level. Subsequently, it is somewhat difficult to track progress over time or determine the level to which a student is performing in real terms.

To overcome this issue, the Grattan Institute employed national NAPLAN data to determine a typical student’s growth trajectory then mapped NAPLAN scale scores onto this trajectory over the school years to establish a series of equivalent year level^1^ (EYL) values (Goss & Sonnemann, 2016). Using these values, it is possible to determine the approximate grade level a student is functioning at from their scale score. For example, a NAPLAN scale score of 476 for reading is the equivalent of a typical student’s performance at the beginning of Year 4. Furthermore, determining a student’s progress between two tests is as simple as subtracting their EYL value on the first from their EYL value on the second. For example, if a student who scored 476 on the Year 5 NAPLAN reading test went on to score 502 on the Year 7 test, they would be achieving at a similar level to typical students at the start of Year 5 and would have made one year of progress in the two years between tests. To allow the use of this method by other researchers, the Grattan Institute provides an Excel spreadsheet indicating the EYL value for any NAPLAN scale score between 100 and 700 (i.e. up to Year 13 standard) on the five NAPLAN tests.

#### Calculating gender gaps in reading and numeracy

In this paper, EYL values were used to convert publicly available NAPLAN reading and numeracy scores for male and female students into equivalent year levels that could be compared across the tested year levels. This comparison sought to determine whether gender gaps exist, their extent, and any patterns in their trajectory over time. The NAPLAN scores were accessed from annual NAPLAN reports from 2008 to 2021. The 2021 report was the most recently available at the time of writing. To determine EYL values using the Grattan Institute’s spreadsheet, any NAPLAN scale scores with decimal places were rounded to whole numbers. Since resulting EYL values were provided up to nine decimal places, these have been rounded to two decimal places in this paper to ease reading. Tables have been constructed to show average NAPLAN mean scores for male and female students in each tested year level between 2008 and 2021, associated EYL values for males and females, and the average gender gap in equivalent years and months. A key advantage of using NAPLAN data and associated EYL values is that NAPLAN tests the whole Australian population rather than a smaller representative sample, which is commonplace in other large-scale tests (e.g. TIMSS, PISA). Analysing the results of the whole population avoids issues of potential selection bias that can lead to distorted pictures of the underlying population subsequently, and it is unnecessary and somewhat misleading to employ inferential statistics (Reilly et al., 2019).

## Findings

### Year 3 reading and numeracy achievement over time

Table 1 shows that, on average, female students achieved higher than male students on the NAPLAN reading test. The average gender gap between 2008 and 2021 for reading was 0.35 years (4.26 months) of equivalent learning. By contrast, males outperformed females on the NAPLAN numeracy test, with an average gender gap across the tested years of 0.14 years (1.71 months) of learning.

**Table 1:** Average Year 3 male and female student reading and numeracy mean scores and EYL values, 2008–2021

Test	Average Year 3 male mean score	Year 3 male EYL	Average Year 3 female mean score	Year 3 female EYL	Average gender gap in years (months)
Reading	413.85	2.62	430.25	2.98	M < F 0.36 yrs (4.25 mths)
Numeracy	404.38	2.93	396.47	2.79	M > F 0.14 yrs (1.71 mths)

**Fig. 2:**
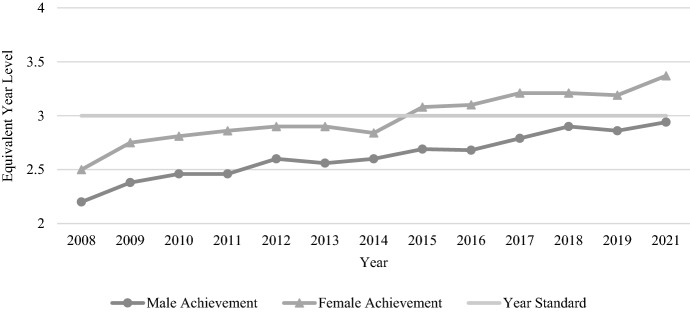
Year 3 reading achievement by gender, 2008–2021

As shown in Fig. 2, females outperformed males on every NAPLAN reading test. Aside from one or two years (e.g. 2014), the performance of both genders increased or decreased each year in a similar way. Importantly, the overall trend with Year 3 reading is one of improvement for both genders.

**Fig. 3:**
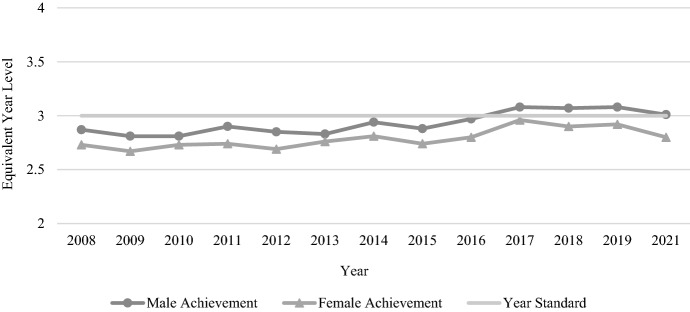
Year 3 numeracy achievement by gender, 2008–2021

Figure 3 shows student performance on each NAPLAN numeracy test, with only a slight improvement evident since NAPLAN began in 2008. Males outperformed females every year; however, the average gender gap is less than half that of reading, at only 0.14 years (1.71 months) of equivalent learning. In this sense, the performance of both genders on the Year 3 test have remained relatively similar over time, with slight improvement.

### Year 5 reading and numeracy achievement over time

**Fig. 4:**
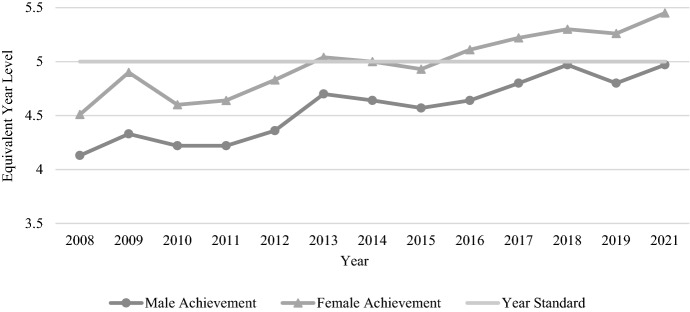
Year 5 reading achievement by gender, 2008–2021

A similar overall picture of improvement is evident in the Year 5 NAPLAN reading results, with both genders increasing their average performance by approximately seven and a half months of equivalent learning between 2008 and 2021 (see Fig. 4). Females once again outperformed males on each test, with the average gender gap increasing slightly from the Year 3 figure to 0.42 years (5.02 months) (see Table 2).

**Table 2:** Average Year 5 male and female student reading and numeracy mean scores and EYL values, 2008–2021

Test	Average Year 5 male mean score	Year 5 male EYL	Average Year 5 female mean score	Year 5 female EYL	Average gender gap in years (months)
Reading	492.44	4.57	505.18	4.98	M < F 0.42 yrs (5.02 mths)
Numeracy	494.93	4.9	484.2	4.61	M > F 0.28 years (3.4 months)

**Fig. 5:**
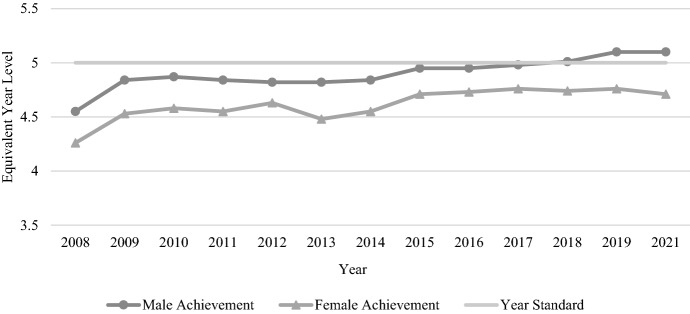
Year 5 numeracy achievement by gender, 2008–2021

While both genders performed at a similar level with numeracy in Year 3, the Year 5 results indicate a clearer gender gap, with males outperforming females (see Fig. 5). The gap of just 0.14 years (1.71 months) in Year 3 doubled in Year 5 to 0.28 years (3.4 months) (see Table 2). The Year 5 results also show stronger student improvement over the years of testing, with both genders in 2021 performing approximately six months of learning ahead of their 2008 counterparts. Scores for both genders increased or decreased in a similar pattern in most years aside from 2012 when there was a clear decline in female scores.

### Year 7 reading and numeracy achievement over time

**Fig. 6:**
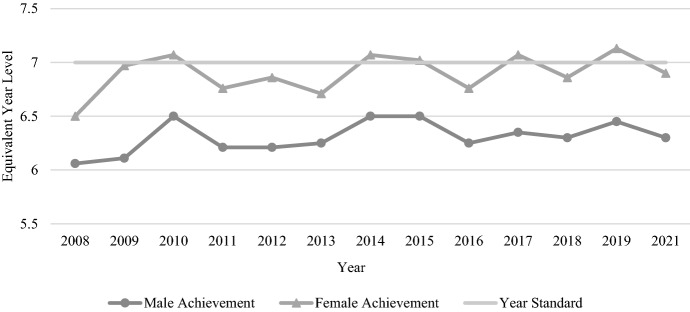
Year 7 reading achievement by gender, 2008–2021

As shown in Table 3, the average gender gap for reading at Year 7 across all NAPLAN reading tests increased from the Year 5 figure to 0.6 years (7.16 months) with females again achieving higher results than males. The Year 7 reading results were notably less consistent than the two tested primary year levels, with scores for both genders creating zigzag patterns between 2008 and 2021, particularly for females (see Fig. 6). While the performance of females in 2021 was approximately five months of learning ahead of their 2008 counterparts, the 2021 result was lower than several other tests (i.e. 2009, 2010, 2014, 2015, 2017, 2019). Results for male students were slightly more consistent over time, with performance spikes in 2010, 2014, 2015, and 2019.

**Table 3:** Average Year 7 male and female student reading and numeracy mean scores and EYL values, 2008–2021

Test	Average Year 7 male mean score	Year 7 male EYL	Average Year 7 female mean score	Year 7 female EYL	Average gender gap in years (months)
Reading	537.01	6.3	548.53	6.9	M < F 0.6 yrs (7.16 mths)
Numeracy	551.39	6.86	541.69	6.47	M > F 0.39 years (4.7 months)

**Fig. 7:**
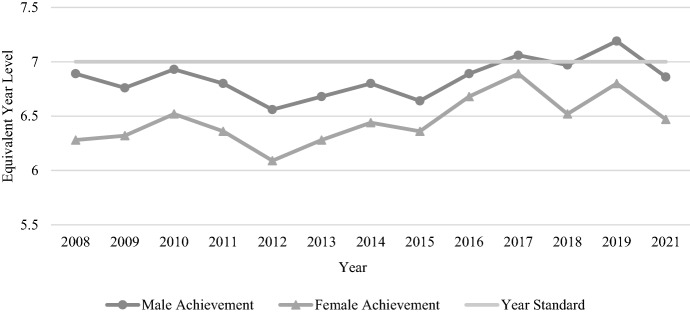
Year 7 numeracy achievement by gender, 2008–2021

The average gender gap for numeracy in Year 7 was 0.39 years (4.7 months). A zigzag pattern was also found in these results, this time for both genders (see Fig. 7). Males once more outperformed females in every NAPLAN numeracy test, though the gap between genders ranged from only 2 months in 2017 to 7.3 months in 2008. The overall trend for females was one of slightly increased performance, despite with notable periods of decline (e.g. 2010–2012) and improvement (2013–2017). Male outcomes in 2021 were slightly below the initial 2008 results.

### Year 9 reading and numeracy achievement over time

**Fig. 8:**
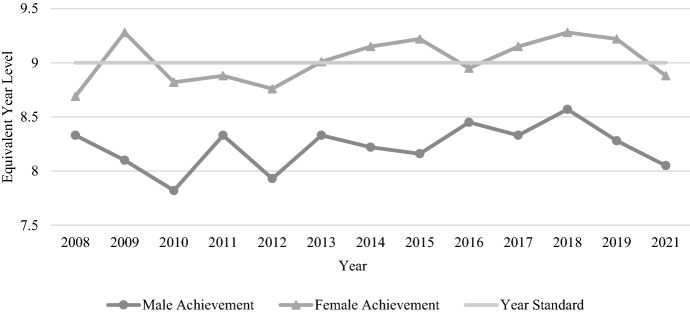
Year 9 reading achievement by gender, 2008–2021

The average gender gap for Year 9 reading across the tested years was 0.8 years (9.56 months) of equivalent learning (see Table 4). These results were unique, in that male and female trends over time seemed quite different (see Fig. 8). While female performance spiked in 2009 to reach the highest score for any female cohort (so much so that we checked the numbers twice), the other years presented a generally consistent picture of improvement (despite notable declines in 2012 and 2016). By contrast, male performance was quite haphazard, increasing or decreasing by several equivalent months of learning from one cohort to the next. Occasionally, scores for both genders increased or decreased together, but these years were exceptions; most of the time, the genders moved in opposite directions. As a result, the gender gap grew and shrunk repeatedly, with the smallest gap being 4.3 months in 2008 and the largest gap being a staggering 14.2 months one year later in 2009.

**Table 4:** Average Year 9 male and female student reading and numeracy mean scores and EYL values, 2008–2021

Test	Average Year 9 male mean score	Year 9 male EYL	Average Year 9 female mean score	Year 9 female EYL	Average gender gap in years (months)
Reading	573.05	8.22	585.88	9.02	M < F 0.8 yrs (9.59 mths)
Numeracy	592.73	8.82	582.92	8.3	M > F 0.52 yrs (6.27 mths)

**Fig. 9:**
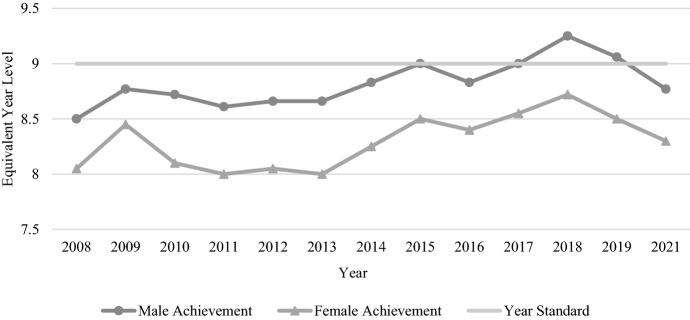
Year 9 numeracy achievement by gender, 2008–2021

Compared to the Year 9 reading results, Year 9 numeracy presented a more consistent picture in terms of performance over time for both genders (see Table 4). Male students outperformed females each year, and scores for the two genders increased or decreased in similarity over time. The smallest gender gap was just 3.8 months, occurring in 2009, while the largest gap occurred in 2013, with 7.9 months between the genders. The average gender gap for Year 9 numeracy across all tested years was 0.52 years (6.27 months) of equivalent learning. There was a relatively clear picture of improvement across the years of NAPLAN testing, with male and female students in 2021 performing approximately 3 months of learning ahead of their counterparts in 2008 (see Fig. 9).

### Other observations from the data

The analysis found evidence of particularly stronger cohorts in both genders. With a focus on reading, for example, the female cohort that completed Year 3 in 2015, Year 5 in 2017, and Year 7 in 2019 performed well above the previous female cohort on the reading test. Similarly, reading scores for the male cohort that completed Year 3 in 2010, Year 5 in 2012, Year 7 in 2014, and Year 9 in 2016 were always higher than the previous male cohort. Having stronger (or weaker) cohorts in each gender meant the gender gap fluctuated in each test and each tested year level. This fluctuation was considerably more apparent in the secondary school years.

In all year levels, there was a marked increase in student performance between 2008 and 2009, particularly for female students. This may be due to school leaders, teachers, and students being more familiar with the NAPLAN test and possibly modifying their practices for the second round of testing.

### Average reading and numeracy achievement by gender over time

**Fig. 10:**
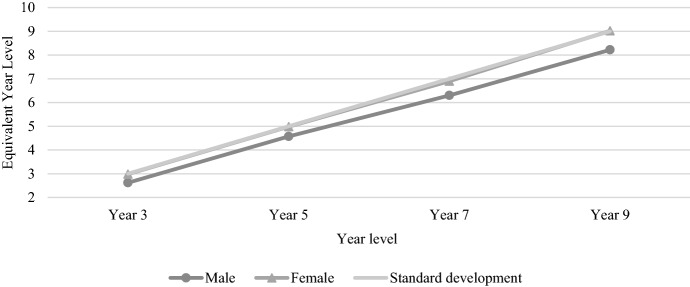
Average reading achievement by gender, 2008–2021

Figure 10 shows the average female and male-reading test performance across the four tested year levels between 2008 and 2021. Female performance improved consistently from Year 3 to Year 9. By contrast, male performance increased at a similar rate to females between Year 3 and Year 5 (resulting in a similar gender gap for these primary year levels), while males fell behind at a faster rate between Year 5 and Year 7. On average, females made approximately two years of progress between each test, but actually made most progress between the Year 7 and Year 9 tests (i.e. 2.12 years). Males made 1.95 years of progress between Year 3 and Year 5 and 1.92 years between Year 7 and Year 9, but only managed 1.73 years of progress between Year 5 and Year 7 (i.e. the transition between primary and secondary school).

**Fig. 11:**
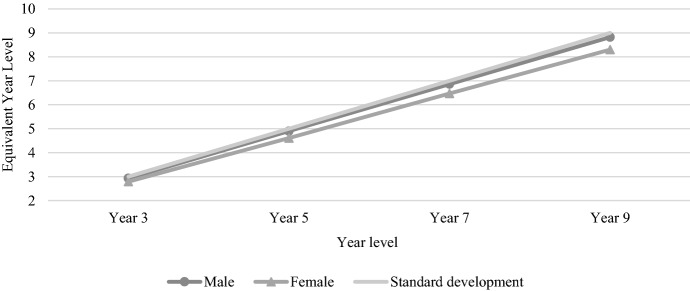
Average numeracy achievement by gender, 2008–2021

Figure 11 shows the average numeracy achievement by gender between 2008 and 2021. The overall picture is quite similar to reading, despite with males performing above females and the gender gap increasing across each year level. Males made approximately two years of progress between each numeracy test, while females consistently made just over 1.8 years of progress between each test, leading to a gender gap that grew wider at a consistent rate over time.

**Fig. 12:**
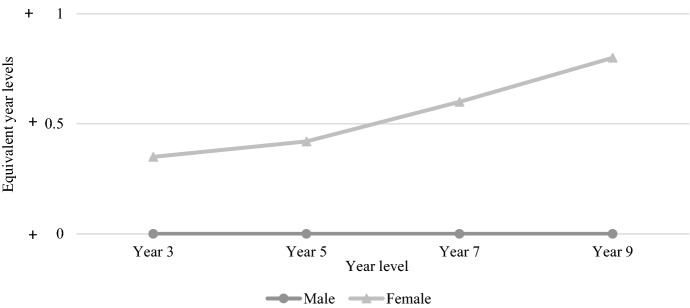
Average gender-based differential for NAPLAN reading, 2008–2021

Figure 12 shows the average gender-based differential across tested year levels between 2008 and 2021 for the reading test. The average gender gap was greater for each increase in year level, with Year 3 males 0.35 years behind Year 3 females, Year 5 males 0.42 years behind Year 5 females, Year 7 males 0.6 years behind Year 7 females, and Year 9 males 0.8 years behind females. While males fell further behind females at every tested year level, the rate at which females outperformed males was greatest between Year 5 and Year 7 and between Year 7 and Year 9. While males made more progress between Year 7 and Year 9, this was also when females made most progress, which explains the seemingly constant rate from Year 5 to Year 9. While males on average keep up with females reasonably well in the primary school years, it seems more males struggle with reading from the transition to secondary school.

**Fig. 13:**
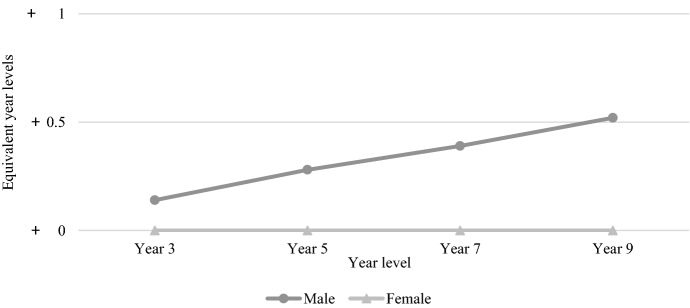
Average gender-based differential for NAPLAN numeracy, 2008–2021

The gender-based differential for NAPLAN numeracy (see Fig. 13) shows a different picture, with females getting further behind with each increase in tested year level. As mentioned above, males and females made consistent progress between each test, though the rate of progress was higher for males, leading to a neatly widening gender gap over time.

## Discussion

Individuals with strong reading and numeracy skills can negotiate communicative and mathematical demands of adult life (Goos et al., 2011; Hochweber & Vieluf, 2018), making their development in the school years a key goal for most teachers. For more than 100 years, researchers have investigated male and female student achievement in these areas (e.g. Hill, 2011; Pickle, 1998), with findings that highlight the complexity of reading and numeracy development. In broad terms, consistent gender gaps have been identified, with females outperforming males in reading and males outperforming females in numeracy. What has been lacking is a clear picture of the developmental trajectory of these gender gaps across the primary and secondary year levels.

The present study converted the publicly available NAPLAN data using EYL values (Goss & Sonnemann, 2016, 2018) to show how Australian male and female students have performed on the NAPLAN reading and numeracy tests since testing began. While others have drawn on the NAPLAN scores to show student performance on NAPLAN testing over time (e.g. Leder & Forgasz, 2018), converting these scores into EYL values took into account the non-linear rate of student progress across the tested year levels, providing a more accurate description of the reading and numeracy gender gaps.

**Fig. 14:**
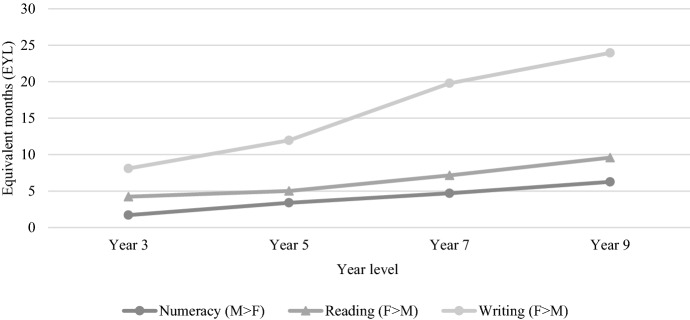
Gender gaps in numeracy, reading, and writing 2008–2021

Thomas (2020) used the NAPLAN writing results and EYL values to provide the first longitudinal picture of male and female achievement on the writing test over time, finding that the average male student performed 8.16 months of equivalent learning behind the average female student in Year 3, 11.88 months behind in Year 5, 20.06 months behind in Year 7, and 24.08 months behind in Year 9. These findings showed that “while boys fell further behind girls at every tested year level, the rate at which girls outperformed boys was greatest between Years 5 and 7” (Thomas, 2020, p. 788). The present study is the first to do the same for the areas of reading and numeracy, finding that similar gaps exist, despite not to the same extent as writing. For ease of comparison, Fig. 14 draws on the findings of this study and Thomas (2020) to show the extent and development of the gender gaps in numeracy, reading, and writing.

The analysis of the reading results showed a fairly similar developmental pattern to that of the writing gender gap, with a notable widening of the gap between Year 5 and Year 7. Female students performed consistently from Year 3 to Year 9, with the average student making approximately two years of progress between each reading test. Previous research has shown that females are more likely to score higher on reading tests and are more likely to be in advanced reading groups at school (Hek et al., 2019), while those who fall below the minimum standards for reading are more likely to be males (Reilly et al., 2019). The results of this study are consistent with such reports, finding that differences in reading achievement were prevalent between the genders. International assessments of students’ reading achievement such as PIRLS and PISA (Lynn & Mikk, 2009) have found that gender differences in reading are universal, with girls from all participating countries significantly and meaningfully outperforming boys.

As revealed by the analysis, males made less progress than females between each reading test, yet they made the least progress between the Year 5 and Year 7 tests (i.e. 1.78 years). This adds to the findings of Thomas (2020) in suggesting that, in terms of literacy achievement, the transition from primary to secondary school is more problematic for male students in both reading and writing. For most Australian students, Year 7 marks the beginning of secondary school, when they will move physically from their primary school campus to a secondary school campus. This physical transition has been shown to impact student reading achievement (see Hopwood et al., 2017). As some students transition from primary school into secondary school, their reading achievement stalls, or in serious cases, decline to levels below that of their primary school years (Hanewald, 2013). In some cases, students entering secondary school have failed to acquire the necessary and basic reading skills in primary school required for secondary school learning (Lonsdale & McCurry, 2004) impacting their ongoing reading development (Culican, 2005). The secondary school curriculum is more demanding; students are expected to be independent readers, able to comprehend a range of complex texts (Duke et al., 2011; Hay, 2014). Heller and Greenleaf (2007) argued that schools cannot settle for a modest level of reading instruction, given the importance of reading for education, work, and citizenship. Due to the importance of reading for an individual’s success and wellbeing, it is critical to understand why this stage of schooling is problematic for many males and how they can be better supported.

The analysis of the numeracy gender gap was quite different from both the reading and writing results. While previous international studies have suggested that the numeracy gender gap only becomes apparent in the secondary school years (Heyman & Legare, 2004), this study showed that average scores for male students were higher than those of female students on every NAPLAN numeracy test, despite to a lesser extent than the other tests. The widest numeracy gender gap of 6.32 months in Year 9 was smaller than the smallest writing gender gap of 8.16 months in Year 3.

Unlike the other tests, male and female students progressed on the numeracy test at more consistent rates across the year levels, though males’ progress of approximately two years between each test was slightly higher than the approximate 1.8 years for females. This difference led to a gradually increasing gender gap.

## Implications

The present study has several implications for theory, research methods, and teacher practice. In terms of theory, the findings highlight links between writing development (Thomas, 2020) and reading development, in that male students appear to find the transition from primary to secondary school particularly challenging. While other researchers have looked at the numeracy gap over time using NAPLAN scale scores (e.g. Leder & Forgasz, 2018), by using EYL values, this study provides a more accurate picture of the gender gap, which increases gradually from the equivalent of 1.71 months of learning in Year 3 to 6.27 months of learning in Year 9. While this supports the general argument that, on average, males outperform females in numeracy and females outperform males in literacy (i.e. reading and writing) tests, it also shows that the gaps are not equal. The literacy gaps are considerably wider, particularly for writing. Female literacy performance does not appear to be affected in the transition from primary to secondary school, while many more males struggle to meet the increased literacy demands of the secondary years (Christie & Derewianka, 2008).

Does this mean sweeping generalisations should be made about male and female student abilities on literacy and numeracy tasks, as has tended to occur in media reports based on NAPLAN results each year (e.g. Bolton, 2019)? In considering detailed studies by Leder and Forgasz (2018) and Cobb-Clark and Moschion (2017), such generalisations are often unhelpful. These authors have found many factors that influence a student’s performance on a standardised reading or numeracy assessment. In certain contexts, females outperform males on these tests, while in others, the results are reversed. There is significant variation in achievement within both genders (ACARA, 2021), and the design of test items may unfairly favour males or females (e.g. Leder & Forgasz, 2018). This suggests that *bigger picture* research in gender gaps that groups males together and females together without considering factors like SES—such as the present study—should be complemented by more focussed research like that by Cobb-Clark and Moschion (2017), which may only explore student achievement at one year level (i.e. Year 3) but comprehensively.

In terms of research methods, international studies into male and female student achievement on standardised assessments have used convenience sampling or other methods of sampling coupled with statistical procedures to predict gender gaps for full populations (e.g. Reilly et al., 2019). By contrast, the NAPLAN assessments are whole-of-population tests, and so allow for more accurate descriptions of performance by different student groups. Given the considerable financial and resourcing costs of NAPLAN implementation, it would be useful for additional studies to explore student performance on these tests over time, particularly since the tests break down scores into demographic factors other than gender (i.e. Indigenous status, language background, geolocation, parental education, and parental occupation).

It is also potentially useful for school leaders and teachers to know that key points in schooling are more difficult for different student groups. According to ACARA (2016d), the first key aim of NAPLAN is to “help drive improvements in student outcomes” (para. 4). Knowing what these tests are broadly revealing about the achievement of different groups of students is a necessary first step for this sort of improvement to be possible. Although broad in focus, the results of this study suggest specific attention may need to be given to supporting male-reading performance in the transition to secondary school. Similarly, while there was no notable year level that females struggled more with numeracy testing, the results suggest that all primary and secondary school teachers may wish to give attention to lifting general female performance in numeracy. A challenge for researchers and teachers is to identify the precise nature of gender differences in reading and numeracy so that teachers can design targeted interventions to ensure gender equality in these vital areas.

## Footnotes

1 An in-depth explanation of the method employed by the Grattan Institute to develop EYL values is beyond the scope of this paper. Should more information be required, the reader is directed to either Goss and Sonnemann (2016) or Goss and Chisholm (2016), the latter providing a broad overview of the mathematics employed to achieve the data transformation.

## References

[CR1] Adams A-M, Simmons FR (2019). Exploring individual and gender differences in early writing performance. Reading and Writing.

[CR2] Australian Curriculum, Assessment and Reporting Authority. (2016a). *Reading*. https://www.nap.edu.au/naplan/reading

[CR4] Australian Curriculum, Assessment and Reporting Authority. (2016b). *Numeracy*. https://www.nap.edu.au/naplan/numeracy

[CR6] Australian Curriculum, Assessment and Reporting Authority. (2016c). *NAPLAN 2012–2016b test papers and answers*. https://bit.ly/3e4qyMQ

[CR7] Australian Curriculum, Assessment and Reporting Authority. (2016d). *Why NAPLAN?* https://www.nap.edu.au/about/why-nap

[CR8] Australian Curriculum, Assessment and Reporting Authority. (2017). *What is numeracy?* https://bit.ly/3q0UNu0

[CR9] Australian Curriculum, Assessment and Reporting Authority. (2021). *NAPLAN national report for 2021*. https://bit.ly/3q6NaC4

[CR10] Australian Curriculum, Assessment and Reporting Authority. (2022). *NAPLAN gets people talking…* https://bit.ly/3dLNQKI

[CR11] Berman, I. (2009). *Supporting adolescent literacy achievement.* https://bit.ly/2T5h7FZ

[CR12] Berninger V, Whitaker D, Feng Y, Swanson H, Abbott R (1996). Assessment of planning, translating, and revising in junior high writers. Journal of School Psychology.

[CR13] Bolton, R. (2019, April 18). Boys’ inability to write clearly is a new crisis in education. Financial Review. *Australian Financial Review.* https://bit.ly/3mbNjTT

[CR14] Caponera E, Sestito P, Russon PM (2016). The influence of reading literacy on mathematics and science achievement. The Journal of Educational Research.

[CR15] Carmichael, C. (2014). Gender, parental beliefs and children’s mathematical performance: Insights from The Longitudinal Study of Australian Children. In J. Anderson, M. Cavanagh & A. Prescott (Eds.), *Proceedings of the 37th annual conference of the mathematics education research group of Australasia* (pp. 119–126). MERGA.

[CR16] Chetty R, Friedman JN, Rockoff JE (2014). Measuring the impacts of teachers II: Teacher value-added and student outcomes in adulthood. American Economic Review.

[CR17] Christie F, Derewianka B (2008). School discourse.

[CR18] Cobb-Clark D, Moschion J (2017). Gender gaps in early educational achievement. Journal of Population Economics.

[CR19] Culican, S. J. (2005). *Learning to read: Reading to learn—A middle years literacy intervention research project. Final Report 2003–4.* Catholic Education Office.

[CR20] Duke NK, Pearson PD, Strachan SL, Billman AK, Samuels SJ, Farstrup AE (2011). Essential elements of fostering and teaching reading comprehension. What research has to say about reading instruction.

[CR21] Education Council. (2019). *Alice Springs (Mparntwe) education declaration*. https://bit.ly/3AI56cZ

[CR22] Evans D, Hatisaru V, Williamson J (2021). The use of NAPLAN data and support for it: Perceptions of practicing teachers. Australian Educational Leader.

[CR23] Forgasz HJ, Hill JC (2013). Factors implicated in high mathematics achievement. International Journal of Science and Mathematics Education.

[CR24] Goos M, Dole S, Geiger V (2011). Improving numeracy education in rural schools: A professional development approach. Mathematics Education Research Journal.

[CR25] Goss, P., & Chisholm, C. (2016). *Widening gaps: What NAPLAN tells us about student progress—Technical Report*. https://bit.ly/3pDkYXG

[CR26] Goss, P., & Sonnemann, J. (2016). *Widening gaps: What NAPLAN tells us about student progress*. https://bit.ly/2IOXzyo

[CR27] Goss, P., & Sonnemann, J. (2018). *Measuring student progress: A state-by-state report card*. https://bit.ly/2UVNxy5

[CR28] Graham S, Herbert M (2011). Writing to read: A meta-analysis of the impact of writing and writing instruction on reading. Harvard Educational Review.

[CR29] Hanewald R (2013). Transition between primary and secondary school: Why it is important and how it can be supported. Australian Journal of Teacher Education.

[CR30] Hardy I, Lewis S (2018). Visibility, invisibility, and visualisation: The danger of school performance data. Pedagogy, Culture & Society.

[CR31] Hatisaru, V. (2020). “[He] has impaired vision due to overworking”: Students’ views about mathematicians. In C. Andrà, D. Brunetto & F. Martignone (Eds.), *Theorizing and measuring affect in mathematics teaching and learning* (pp. 89–100). Springer. 10.1007/978-3-030-50526-4_9

[CR32] Hatisaru V (2021). Theory-driven determinants of school students’ STEM career goals: A preliminary investigation. European Journal of STEM Education.

[CR33] Hay I, Fitzallen N, Reaburn R, Fan S (2014). Literacy development: An interactive perspective. The future of educational research: Perspectives from beginning researchers.

[CR34] Hek M, Buchman C, Kraaykamp G (2019). Educational systems and gender differences in reading: A comparative multilevel analysis. European Sociological Review.

[CR35] Heller, R., & Greenleaf, C. (2007). *Literacy instruction in the content areas: Getting to the core of middle and high school improvement.* Alliance for Excellent Education.

[CR36] Heyman GD, Legare CH (2004). Children’s beliefs about gender differences in the academic and social domains. Sex Roles.

[CR37] Hill, J. C. (2011). Gender differences in NAPLAN mathematics performance. In J. Clark, B. Kissane, J. Mousley, T. Spencer & S. Thornton (Eds.), *Mathematics: Traditions and [New] practices. Proceedings of the 34th annual conference of the Mathematics Research Group of Australasia* (pp. 366–372). MERGA.

[CR38] Hochweber J, Vieluf S (2018). Gender differences in reading achievement and enjoyment of reading: The role of perceived teacher quality. The Journal of Educational Research.

[CR39] Hopwood B, Hay I, Dyment J (2017). Students' reading achievement during the transition from primary to secondary school. Australian Journal of Language and Literacy.

[CR40] Jackson CJ (2022). The utility of NAPLAN data: Issues of access, use and expertise for teaching and learning. Australian Journal of Language and Literacy.

[CR41] Kane JM, Mertz JE (2012). Debunking myths about gender and mathematics performance. Notices of the American Mathematical Society.

[CR42] Khorramdel L, Pokropek A, Joo S-H, Kirsch I, Halderman L (2020). Examining invariance approach. Psychological Test and Assessment Modelling.

[CR43] Leder, G. C., & Forgasz, H. (2011). The public’s views on gender and the learning of mathematics: Does age matter? In J. Clark, B. Kissane, J. Mousley, T. Spencer, & S. Thornton (Eds.), *Mathematics: Traditions and [New] practices. Proceedings of the 34th annual conference of the Mathematics Research Group of Australasia* (pp. 446–545). MERGA.

[CR44] Leder GC, Forgasz H (2018). Measuring who counts: Gender and mathematics assessment. ZDM.

[CR45] Leder GC, Forgasz HJ, Jackson G (2014). Mathematics, English and gender issues: Do teachers count?. Australian Journal of Teacher Education.

[CR46] Lee JAC, Al Otaiba S (2015). Socioeconomic and gender group differences in early literacy skills: A multiple-group confirmatory factor analysis approach. Educational Research and Evaluation.

[CR47] Lewis S, Hardy I (2015). Funding, reputation and targets: The discursive logics of high-stakes testing. Cambridge Journal of Education.

[CR48] Logan S, Johnston R (2010). Investigating gender differences in reading. Educational Review.

[CR49] Lonsdale, M., & McCurry, D. (2004). *Literacy in the new millennium.* Australian Government, Department of Education, Science and Training.

[CR50] Lynn R, Mikk J (2009). Sex differences in reading achievement. Trames.

[CR52] McGeown S, Goodwin H, Henderson N, Wright P (2012). Gender differences in reading motivation: Does sex or gender identity provide a better account?. Journal of Research in Reading.

[CR53] McKenna MC, Conradi K, Lawrence C, Jang BG, Meyer JP (2012). Reading attitudes of middle school students: Results of a U.S. survey. Reading Research Quarterly.

[CR51] Ministerial Council on Education, Employment, Training and Youth Affairs. (2008). *The Melbourne declaration on educational goals for young Australians*. https://bit.ly/3cbLBzR

[CR54] Neufeld P (2006). Comprehension instruction in content area classes. The Reading Teacher.

[CR55] Oam, J. D. L. (2015). *Why does gender matter?* (Unpublished master’s thesis). Monash University, Melbourne, VIC, Australia.

[CR56] Organisation for Economic Co-operation and Development. (2012). *Literacy, numeracy and problem solving in technology-rich environments: Framework for the OECD survey of adult skills*. OECD.

[CR57] Organisation for Economic Co-operation and Development. (2020). *“Girls’ and boys’ performance in PISA” in PISA 2018 results (VOLUME 2)” Where all students can succeed.* OECD Publishing.

[CR58] Partanen M, Siegel LS (2014). Long-term outcome of the early identification and intervention of reading disabilities. Reading and Writing.

[CR59] Pauley FR (1951). Sex differences and legal school entrance. The Journal of Educational Research.

[CR60] Picker S, Berry J (2000). Investigating pupils images of mathematicians. Educational Studies in Mathematics.

[CR61] Pickle JM (1998). Historical trends in biological and medical investigations of reading disabilities: 1850–1915. Journal of Learning Disabilities.

[CR62] Pinkett M, Roberts M (2019). Boys don’t try? Rethinking masculinity in schools.

[CR63] Reilly D, Neuman D, Andrews G (2019). Gender differences in reading and writing achievement: Evidence from the National Assessment of Educational Progress (NAEP). American Psychologist.

[CR64] Ryan M, Khosronejad M, Barton G, Kervin L, Myhill D (2021). A reflexive approach to teaching writing: Enablements and constraints in primary school classrooms. Written Communication.

[CR65] Scheiber C, Reynolds MR, Hajovsky DB, Kaufman AS (2015). Gender differences in achievement in a large, nationally representative sample of children and adolescents. Psychology in the Schools.

[CR66] Smith R, Snow P, Serry T, Hammond L (2021). The role of background knowledge in reading comprehension: A critical review. Reading Psychology.

[CR67] Thomas DP (2020). Rapid decline and gender disparities in the NAPLAN writing data. The Australian Educational Researcher.

[CR68] Thomson, S., de Bortoli, L., & Buckley, S. (2013). *PISA 2012: How Australia measures up*. Australian Council for Educational Research.

[CR69] Unsworth L, Cope J, Nicholls L (2019). Multimodal literacy and large-scale literacy tests: Curriculum relevance and responsibility. Australian Journal of Language and Literacy.

[CR70] Victorian Curriculum and Assessment Authority. (2013). *Using NAPLAN data diagnostically: An introductory guide for classroom teachers*. https://bit.ly/3R69tnA

[CR71] Wyn, J., Turnbull, M., & Grimshaw, L. (2014). *The experience of education: The impacts of high stakes testing on school students and their families*. https://www.whitlam.org/publications/2017/10/17/the-experience-of-education-a-qualitative-study

